# Immunization with Recombinant Prion Protein Leads to Partial Protection in a Murine Model of TSEs through a Novel Mechanism

**DOI:** 10.1371/journal.pone.0059143

**Published:** 2013-03-15

**Authors:** Konstantinos Xanthopoulos, Rosa Lagoudaki, Anastasia Kontana, Christos Kyratsous, Christos Panagiotidis, Nikolaos Grigoriadis, Minas Yiangou, Theodoros Sklaviadis

**Affiliations:** 1 Laboratory of Pharmacology, School of Pharmacy, Aristotle University of Thessaloniki, Thessaloniki, Greece; 2 Department of Neurology, Laboratory of Experimental Neurology and Neuroimmunology, AHEPA University Hospital, Thessaloniki, Greece; 3 Department of Genetics, Development & Molecular Biology, School of Biology, Aristotle University of Thessaloniki, Thessaloniki, Greece; 4 Laboratory of Cell Biology, School of Pharmacy, Aristotle University of Thessaloniki, Thessaloniki, Greece; The Scripps Research Institute Scripps Florida, United States of America

## Abstract

Transmissible spongiform encephalopathies are neurodegenerative diseases, which despite fervent research remain incurable. Immunization approaches have shown great potential at providing protection, however tolerance effects hamper active immunization protocols. In this study we evaluated the antigenic potential of various forms of recombinant murine prion protein and estimated their protective efficacy in a mouse model of prion diseases. One of the forms tested provided a significant elongation of survival interval. The elongation was mediated via an acute depletion of mature follicular dendritic cells, which are associated with propagation of the prion infectious agent in the periphery and in part to the development of humoral immunity against prion protein. This unprecedented result could offer new strategies for protection against transmissible encephalopathies as well as other diseases associated with follicular dendritic cells.

## Introduction

Transmissible spongiform encephalopathies (TSEs) or Prion diseases are invariably lethal neurodegenerative diseases, afflicting a wide range of hosts, including Creutzfeldt-Jakob disease (CJD) in humans, bovine spongiform encephalopathy (BSE) in cattle and scrapie in sheep and goats [1]. The common pathogen to all these ailments is termed prion and is believed to consist primarily if not exclusively of the disease associated isoform of the PrP protein (PrP^Sc^) [2]. PrP^Sc^ arises from the structural conversion of the cellular isoform of the prion protein (PrP^C^). PrP^C^ is a highly conserved, ubiquitously expressed glycoprotein, whose functions remain elusive [3].

The conversion of the cellular prion protein to the disease-associated isoform is a key point to the disease process, but has not yet been elucidated [4]. The two isoforms share the same primary structure and only differ in their secondary structure; PrP^C^ is α-helices rich, whereas in PrP^Sc^ the percentage of β-pleated sheets is elevated. This structural difference leads to changes in resistance to proteinase K proteolysis (PrP^Sc^ is partially resistant) and solubility (PrP^Sc^ is insoluble) [1].Oxidation of some methionine residues in PrP^Sc^ is the only post translation chemical modification reported [5].

TSEs can be transmitted via a variety of routes; however it is believed that ingestion of the pathogen accounts for the majority of naturally acquired cases [6]. Prion pathogenesis takes place in three distinct phases: following initial peripheral exposure and uptake, the pathogen i) replicates in the periphery, *i.e.* the lymphatic system ii) migrates through the peripheral to the central nervous system and eventually iii) from the central nervous system back to peripheral sites [7].

Despite the fact that the pathogen remains in the periphery for an extended period of time, the immune system is not capable of mounting an efficient response that would eliminate it. Instead there is substantial evidence that some cell types of the immune system might actually facilitate disease progression. Follicular dendritic cells (FDCs), in which the pathogen is believed to proliferate throughout early stages of disease, at least in some types of TSEs [8], [9], are among the most important. In the absence of FDCs, prion propagation can be achieved in different immune cells, including MOMA-1 positive macrophages [10], while a population of lymphotoxin β responsive stromal cells of granulomas has been shown capable of supporting prion proliferation [11]. High endothelial venules, which are required for entry of lymphocytes into lymph nodes were recently found to support entry of the prions to the lymph nodes and thus support prion accumulation in the absence of FDCs [12]. Plasmacytoid dendritic cells and natural killer cells were shown to sustain high infectivity titers, indicating their role in the spread of the disease [13] and B-cells in addition to controlling differentiation of FDCs, through tumor necrosis factor α and lymphotoxin α and β signaling, were associated with dissemination of prions from the FDCs to secondary lymphoid organs [14]. Other studies pointed towards the importance of the complement system for efficient prion transmission [15], [16]. In agreement with all the above, stimulation of the immune system via repetitive administration of CpG facilitates infection in a mouse model of prion diseases [17] and question has arisen as to whether it would be more convenient to suppress rather than stimulate the immune system for prophylaxis [18].

Immune-based approaches for prion prophylaxis have been widely used and remain among the most promising preventive strategies. Early experiments showed that monoclonal anti-PrP antibodies could protect against accumulation of PrP^Sc^ in infected cell lines and actually clear infection [19], [20]. Later it was shown that passive immunization [21] or transgenic expression of anti-PrP antibodies [22] could provide protection in a murine model of prion disease, in which the pathogen was peripherally administered. Active immunization protocols were not similarly successful, leading to extension of the survival interval, rather than prophylaxis [23]–[26]. To date, full protection was only achieved via mucosal vaccination, which was successful in an oral transmission model of prion diseases [27], [28].

In this work, various forms of recombinant murine prion protein, including a fusion protein, a mixture with immunogenic molecules and an aggregated protein suspension, were tested as antigens and the potential protection was assessed in a murine model of prion disease. Furthermore the induced immune response and the immunization effect were evaluated on a number of cell types of the immune system. Our results indicate that it is possible to provide partial protection against prion diseases through active immunization. Importantly, we show that the protective effect is associated with the reduction of mature FDCs and only in part to the production of antibodies capable of recognizing PrP.

## Materials and Methods

### Ethics Statement

Animal experiments in this study were performed in accordance to National (presidential decree 160/91) and institutional requirements, under the supervision of the regional veterinary services, which reviewed and approved them. The animal facility is approved by the local veterinary services (accreditation number EL 54 BIO 29) and the office of laboratory animal welfare (A5931-01).

### Recombinant Proteins and Antigens Production

Plasmids for the production of murine recombinant PrP (mrPrP), recombinant DnaK from *Escherichia coli* (DnaK) and the PrP-DnaK fusion protein (PrP-DnaK) were prepared as detailed in [29] whereas the germacrene β-synthase plasmid was a generous gift from Dr. V. Falara [30]. All the recombinant proteins were produced in transformed BL21(DE3) cells and purified as inclusion bodies [31].

To prepare aggregated PrP (agPrP), purified mrPrP inclusion bodies were suspended in sterile phosphate buffered saline (PBS) containing 0.05% Sarkosyl (Sigma) and the PrP content was estimated by densitometric analysis using Image J (v 1.46, available at http://rsbweb.nih.gov/ij/download.html) following SDS-PAGE and coomassie staining. An identical approach was utilized to produce and purify the germacrene β-synthase aggregates (Ger). Lipopolysaccharide contamination in the purified inclusion bodies was assessed with a commercially available Limulus polyphemus endotoxin detection test (Camprex). To prepare solubilized PrP (sPrP), mrPrP was further purified from the inclusion bodies, using Ni-NTA beads (Qiagen) according to the manufacturer’s instructions. mrPrP elution from the nickel beads was performed using 8 M urea, which was gradually diluted to 1 M. The purified protein that remained soluble in 1 M urea was concentrated, using a centrifugal concentrator with a 10 kDa cutoff (VivaSpin 2,Sartorius biotechnology). DnaK and the PrP-DnaK fusion proteins were also purified using Ni-NTA beads [29].

All vaccinations were performed with 100 µg of protein. The sPrP+DnaK mix was produced by mixing solubilized PrP with DnaK in a 3∶7 (*w/w*) ratio, to mimic the relative abundance of each protein in the PrP-DnaK fusion molecule. The antigens were prepared just before administration to the mice by mixing appropriate volumes of the protein-solutions or suspensions with equal volumes of Freund’s adjuvant (Sigma). Complete Freund’s adjuvant (FA) containing 0.4 mg/ml killed *Mycobacterium tuberculosis* was used for priming, whereas boosts were performed with incomplete FA.

### Animal Experiments

Female C57Bl/6 J mice approximately 8 weeks old were used throughout. Animals were housed in a P3 security level facility at constant temperature (22°C), with a 12 h day-night cycle and provided food and water *ad libitum*. Sheets of paper were used as housing enrichment. The immunization protocol consisted in one priming and two booster subcutaneous administrations, separated by 14 days intervals.

Two types of animal experiments were performed: long- and short-term. In long-term experiments mice were immunized with the mix of sPrP and DnaK (sPrP+DnaK+FA group), solubilized PrP (sPrP+FA group), PrP-DnaK fusion protein (PrP-DnaK+FA group) and aggregated PrP (agPrP+FA group). FA was used across all groups and thus a control group received FA alone. A second control group consisted of naive mice. Seven days after the second booster immunization blood was collected from each individual via retrobulbar bleeding and 3 days later mice were challenged with a mouse adapted scrapie strain (RML). Challenge material consisted of 100 µl of a 1% brain homogenate from terminally ill, RML-infected C57Bl/6 J mice, administered intraperitoneally (10^3.5^ × LD_50_). Challenged mice were kept under constant supervision and sacrificed at terminal stage. No signs of distress were evident until mice reached terminal stage. Terminal stage symptoms included hunched posture, severe weight loss and paralysis. Mice displaying these symptoms were sacrificed by cervical dislocation and their brain and spleen were collected for further analysis.

In short-term experiments mice were immunized as previously described with agPrP alone (agPrP group) or agPrP and FA (agPrP+FA group) whereas the control groups received FA alone (FA group) or left naive. Ten days after the second boost, mice were either sacrificed or challenged with RML as previously described. Spleens were harvested from the sacrificed mice and snap-frozen for immunohistofluorescence or prepared for cell proliferation assays. Challenged mice were sacrificed by cervical dislocation 40 or 80 days post challenge and their brain and spleen harvested for further analysis.

### Western Blotting and ELISA

Solubilized recombinant PrP as well as unprocessed or enriched in PrP^Sc^ [32] brain homogenates from terminally ill mice were analyzed by western blotting. For western blotting, proteins were resolved onto 13% polyacrylamide gels and then electrotransferred onto polyvinylidenefluoride membranes (PVDF, Millipore). Immune sera (diluted 1∶200 in blocking buffer (5% *w/v* non-fat dry milk in PBS containing 0.1% *v/v* Tween 20)) or 6H4 (Prionics, 0.2 µg/ml in blocking buffer) were used as primary antibodies. Bound antibodies were detected with HRP-conjugated anti-mouse IgG (Pierce, 0.1 µg/ml in blocking buffer) and the protein bands visualized on X-Ray films (Fuji), following incubation with the ECL reagent.

For ELISAs, medium binding 96-well plates (Greiner) were coated o/n at 4°C with a sPrP solution (0.1% *w/v* in 0.1 mM NaHCO_3_, pH 9.6) and blocked with blocking buffer (5% *w/v* non-fat dry milk in PBS containing 0.1% *v/v* Tween 20). Immune sera (diluted 1∶100 in blocking buffer (5% *w/v* BSA in PBST)) and control monoclonal antibodies (0.2 µg/ml in blocking buffer) were used as primary antibodies. HRP-conjugated anti-mouse IgG (Pierce, 0.1 µg/ml in blocking buffer) was used to detect the bound antibodies. OD_405_ was measured with an automated plate reader after incubation with an ABTS solution (Sigma, 420 mM in 50 mM citric acid, 0.0516% H_2_O_2_).

### Cell Proliferation Assays

Ten days after the second booster, mice were sacrificed by cervical dislocation and their spleens aseptically collected. After counting splenocytes with a haemocytometer these were cultured (2 × 10^5^ cells/well) in triplicates in 0.2 ml of RPMI-1640 complete medium [33] in the presence or absence of antigen (agPrP: 125 µg/ml; sPrP: 125 µg/ml; Ger: 125 µg/ml) or positive controls (concanavalin A, ConA, 0.5 µg/ml and lipopolysaccharide, LPS, 5 µg/ml) for 72 h at 37°C and 5% *v/v* CO_2_ atmosphere. During the last 18 h, 0.04 µCi tritiated thymidine per well was added, and splenocyte proliferation was determined by tritiated thymidine incorporation using a β-counter.

### Flow Cytometric Analysis

One million DT40 cells stably transfected with murine PrP were transferred to 100 µl PBS containing 0.5% FBS and 1 µl FcR blocker (purified 2.4G2 rat anti-mouse monoclonal, (BD Pharmingen). 0.5 µl of immune sera (1∶200 dilution) or 6H4 (2 µg/ml) were then added and the cells were incubated on ice for 30 minutes, washed twice with PBS containing 0.5% *v/v* FBS, and incubated with 1 µl of the secondary reagent (goat anti-mouse Ig FITC, BD Pharmingen) for 30 minutes on ice. After washing three times with PBS containing 0.5% *v/v* FBS, the cells were transferred to FACS tubes and analyzed on a FACScalibur (BD). Data was processed using Flowjo (v. 9).

### Immunohistofluorescence, Immunohistochemistry and Histopathology

Immunohistofluorescence was used to detect folicular dendritic cells, B-cells, T-cells and macrophages markers on splenic sections from mice sacrificed 10 days after the second booster immunization. Splenic sections (6 µm thick) were prepared from fresh frozen splenic tissue. These were blocked for 1 h in antibody diluent (Dako cytomation) and then incubated for 16 h at 4°C with the primary antibody diluted in antibody diluent. Anti-FDC-M1 (BD Pharmingen, 1∶50 *v/v*), anti-B220/CD45 (BD Pharmingen, 1∶200 *v/v*), anti-CD3 (Neomarkers, 1∶200 *v/v*) and anti-MAC-3 (BD Pharmingen, 1∶400 *v/v*) were used for the detection of FDCs, B-cells, T-cells and macrophages. Bound antibodies were detected with the corresponding secondary antibodies (Invitrogen, 1∶200 *v/v*/), the cell nuclei were counterstained with DAPI (Invitrogen) and the sections were prepared for microscopic observation.

The glial fibrillary acidic protein (GFAP) was used as a marker for astrocytosis. To this end, 10 µm thick mouse brain paraffin sections were deparaffinized using xylene and rehydrated. Antigen retrieval was performed for 1 h in a vegetable steamer using citric acid (0.01 M). The sections were then washed, brought to room temperature, blocked for 1 h with antibody diluent (Dako cytomation) and incubated for 16 h with the primary antibody (Dako, 1∶2000 *v/v* in antibody diluent). Eventually the sections were washed with TBS and incubated for 0.5 h with Envision+ (Dako cytomation), before being washed and incubated with a diaminobenzidine solution.

To detect neuropathological lesions in the brain, the paraffin sections were deparaffinized and incubated for 6 min in a Meyer hematoxylin solution. The membranes were then washed, incubated for 45 seconds in HCl, washed again and incubated for 4 min in an eosin solution.

## Results

### Production and Purification of Recombinant Proteins

Recombinant proteins were expressed in BL21(DE3) cells. The production of the desired protein was confirmed by western blotting, using suitable detection antibodies, whereas the yield and purity of the recombinant proteins was estimated by SDS-PAGE, followed by coomassie staining and densitometric analysis. Purity of recombinant proteins typically exceeded 95%, while the purified inclusion bodies contained the recombinant protein in a ratio not lower than 70%. LPS contamination was barely detectable with the LAL test (data not shown). Typical western blots and electrophoretic analyses for the detection of the recombinant proteins are presented in Sup. Figure 1 and [29], [30].

**Figure 1:**
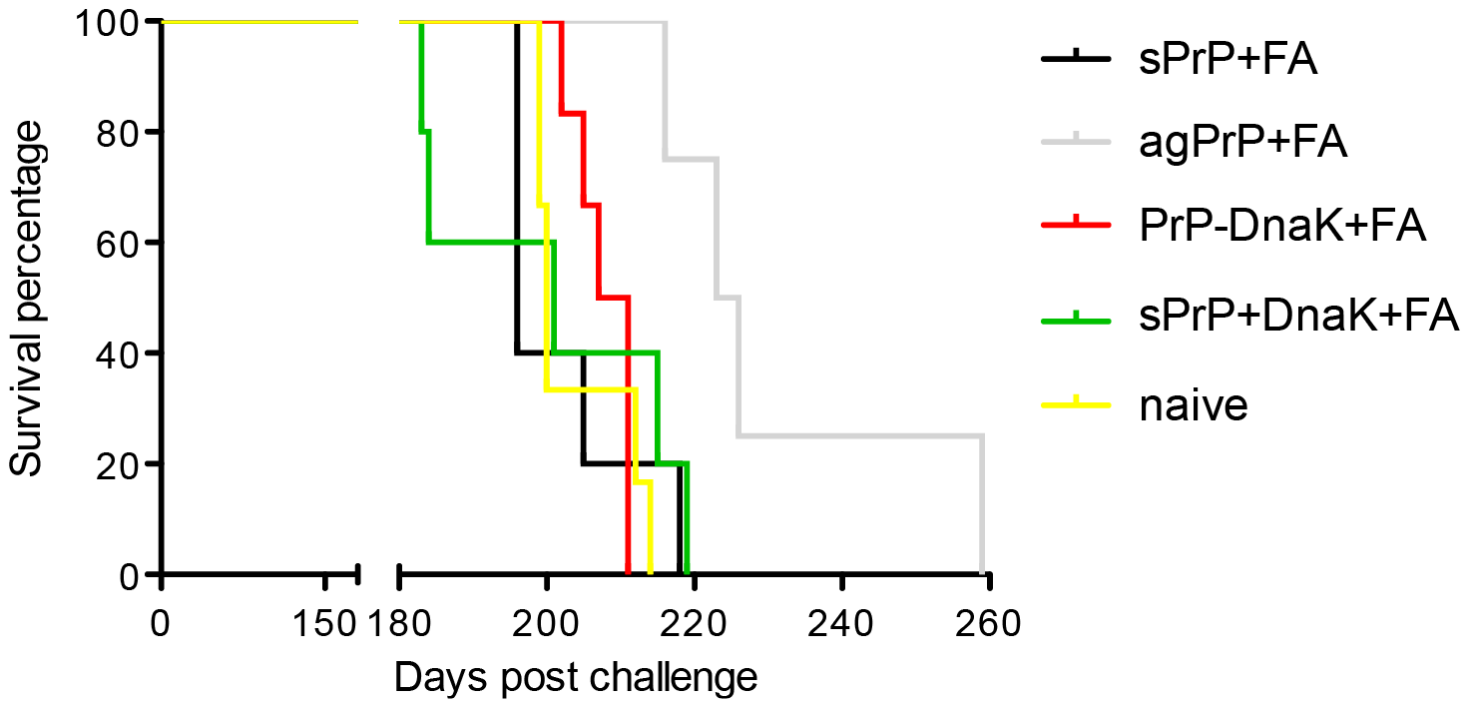
Survival curves of immunized mice. Naive mice and mice immunized with sPrP+FA, agPrP+FA, PrP-DnaK+FA, sPrP+DnaK+FA or FA alone were challenged with a mouse adapted scrapie strain and sacrificed at terminal point. Mice immunized with agPrP+FA survive significantly longer than naive mice (Mantel-Cox test, P = 0.0033).

### Animal Studies

To assess the therapeutic effect the immunization protocol may have, the immunized mice were challenged with a mouse adapted scrapie strain (RML). Three out of five mice immunized with FA alone succumbed before the appearance of TSE-associated symptoms, from causes unrelated to the immunization or challenge and thus the whole group was excluded from the analysis. Survival intervals of the remaining groups were estimated and mice immunized with sPrP+FA, PrP-DnaK+FA or sPrP+DnaK+FA displayed similar survival with naive mice (approximately 200 days). Survival curves of mice immunized with agPrP+FA, however, were significantly different than naive controls (Mantel-Cox test, P = 0.0033), and these mice survived approximately 28 days longer than naive mice (T-test, P = 0.0121) (Figure 1). All terminally ill mice had similar PrP^Sc^ accumulation in their brains, as assessed by western blotting (Sup. Figure 2), and comparable degree of neurological damage and astrocytosis, as evidenced by neuropathological evaluation and immunohistochemistry (Sup. Figure 3).

**Figure 2:**
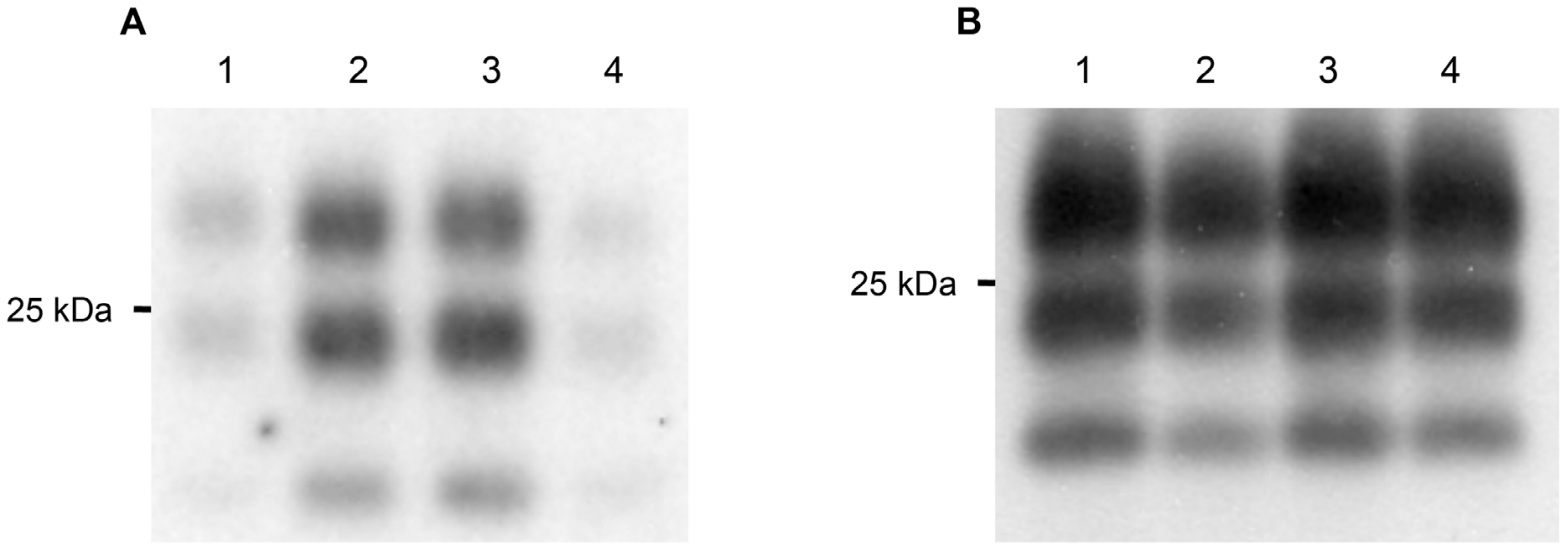
Splenic PrP^Sc^ accumulation. Early PrP^Sc^ accumulation in spleens from naive mice (lane 1), mice immunized with FA alone (lane 2), agPrP alone (3) or agPrP+FA (4), 40 (A) or 80 (B) days after challenge. Splenic samples from 3 mice per group were enriched in PrP^Sc^ and blotted with the polyclonal antibody SAL 1 [39]. Equal amounts of total protein from three mice per group were processed.

**Figure 3:**
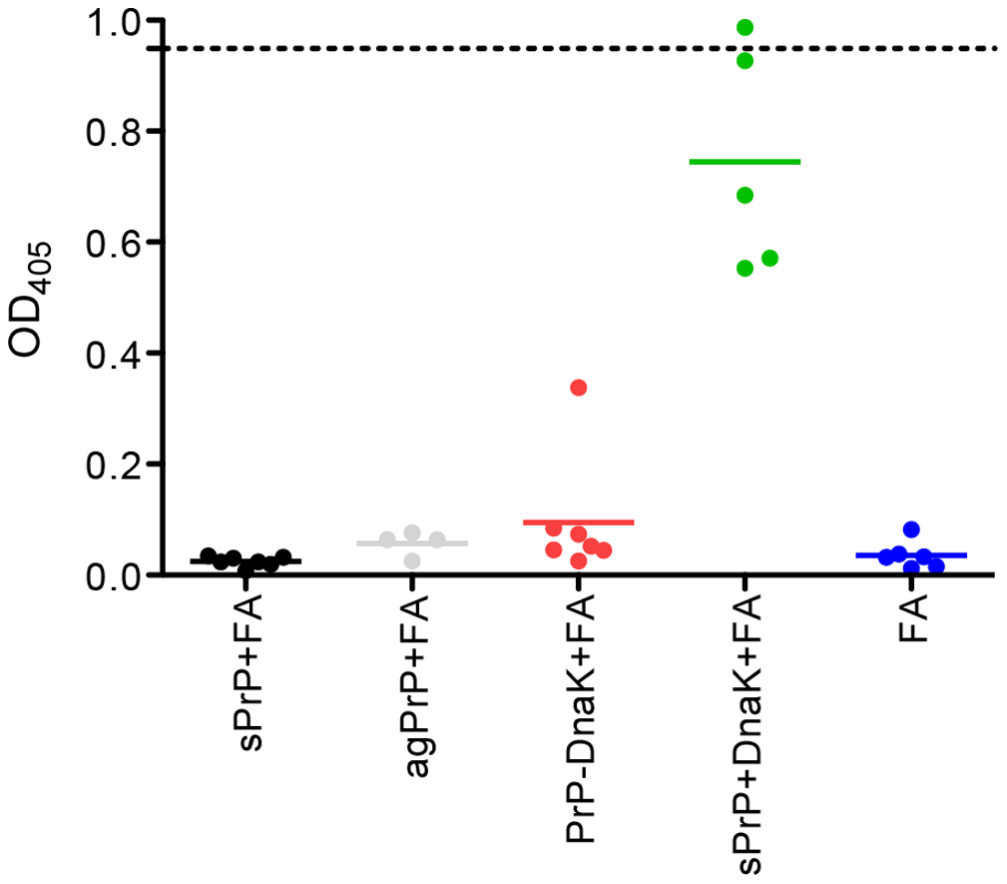
sPrP is recognized with the immune sera in ELISAs. Sera from mice immunized with sPrP+FA, sPrP+DnaK+FA, PrP-DnaK+FA. agPrP+FA or FA alone were tested for their ability to recognize sPrP in ELISA in a 1∶100 (*v/v*) dilution. Each point corresponds to one individual. Line: average value; dotted line OD_405_. of the positive control antibody (6H4, 0.2 µg/ml).

Similar results were obtained, when earlier time points were analyzed. For these experiments focus was given to the agPrP immunized animals, which were checked for splenic PrP^Sc^ accumulation 40 days post challenge. In agreement to our results from the long-term assays, animals immunized with agPrP displayed a lower splenic PrP^Sc^ burden compared to control animals. Densitometric analysis indicates that mice immunized with agPrP+FA accumulate approximately 50% less splenic PrP^Sc^ than their naïve counterparts. On the contrary, 80 days post challenge all animals displayed similar splenic PrP^Sc^ accumulation (Figure 2). In these earlier time points, neuropathological findings and neurodegeneration were not detectable (data not shown).

### Characterization of the Immune Response

#### i) Production of anti-PrP antibodies

**Figure 4:**
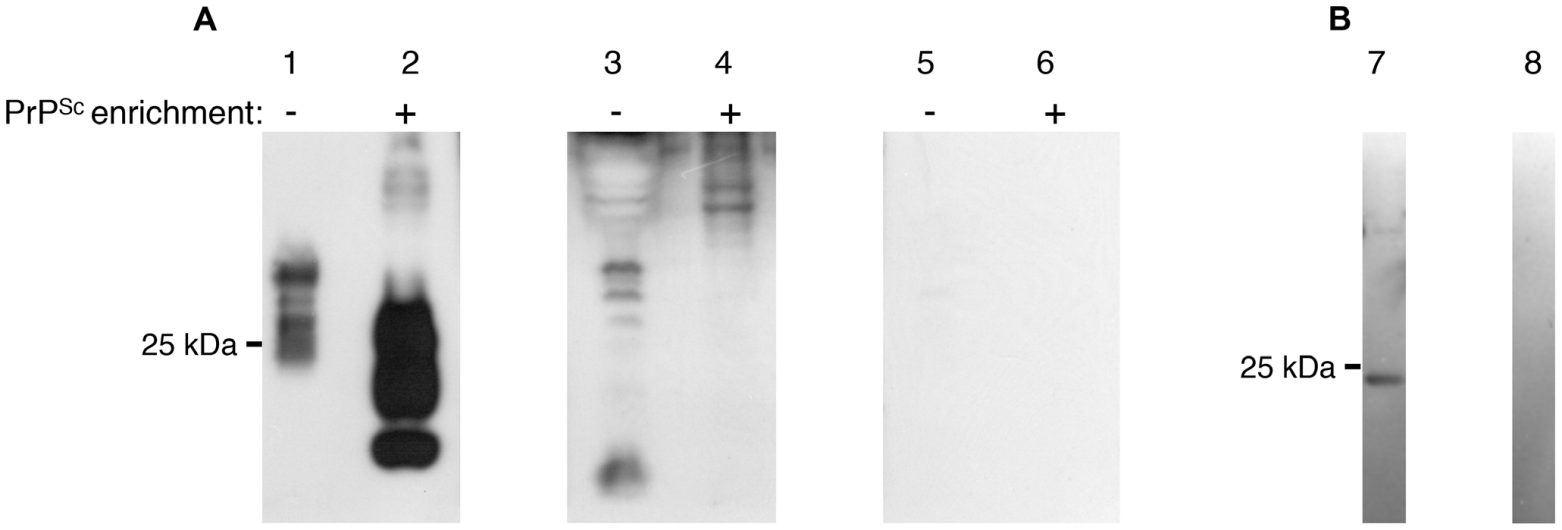
Sera from sPrP+DnaK mice recognize total PrPSc and sPrP in western blots. A. 2.5 mg brain equivalent from terminally ill mice were blotted with 6H4 (lanes 1, 2), serum from a mouse immunized with sPrP+DnaK (lanes 3, 4) or with the secondary antibody alone (lanes 5, 6), prior (lanes 1, 3, 5) or ensuing (lanes 2, 4, 6) PrP^Sc^ enrichment. B. sPrP (1 µg) was blotted with serum from a mouse immunized with sPrP+DnaK (lane 7) or the secondary antibody alone (lane 8).

Sera collected from the immunized mice 7 days after the second booster injection were checked by ELISA for the presence of antibodies specific for PrP. Only sera from mice immunized with the PrP+DnaK mix were capable of recognizing recombinant murine PrP in this setting, whereas all other sera tested negative, including sera from mice immunized with agPrP, which survived longer in the bioassay (Figure 3). Similar results were obtained when the sera were tested by western blotting; sera from PrP+DnaK-immunized mice bound to recombinant murine PrP as well as total PrP in brain homogenates from mice terminally ill with scrapie. The same sera failed to recognize the three-banded pattern associated with PrP^Sc^ in PrP^Sc^-enriched brain homogenates, although some higher apparent molecular weight forms –possibly 6H4-reacting PrP-multimers- were blotted (Figure 4). Sera from mice immunized with agPrP failed to bind PrP in western blots (Sup. Figure 4).

#### ii) Flow cytometry

Sera from sPrP+DnaK+FA- and agPrP+FA-immunized mice were further tested for their ability to bind PrP on the surface of transgenic DT40 cells, which express murine prion protein on the cell surface. Binding of PrP on the cell surface is indicative of the capability of the antibody to recognize native forms of PrP and has been associated with the protective role of the antibody against prion diseases [34].

**Figure 5:**
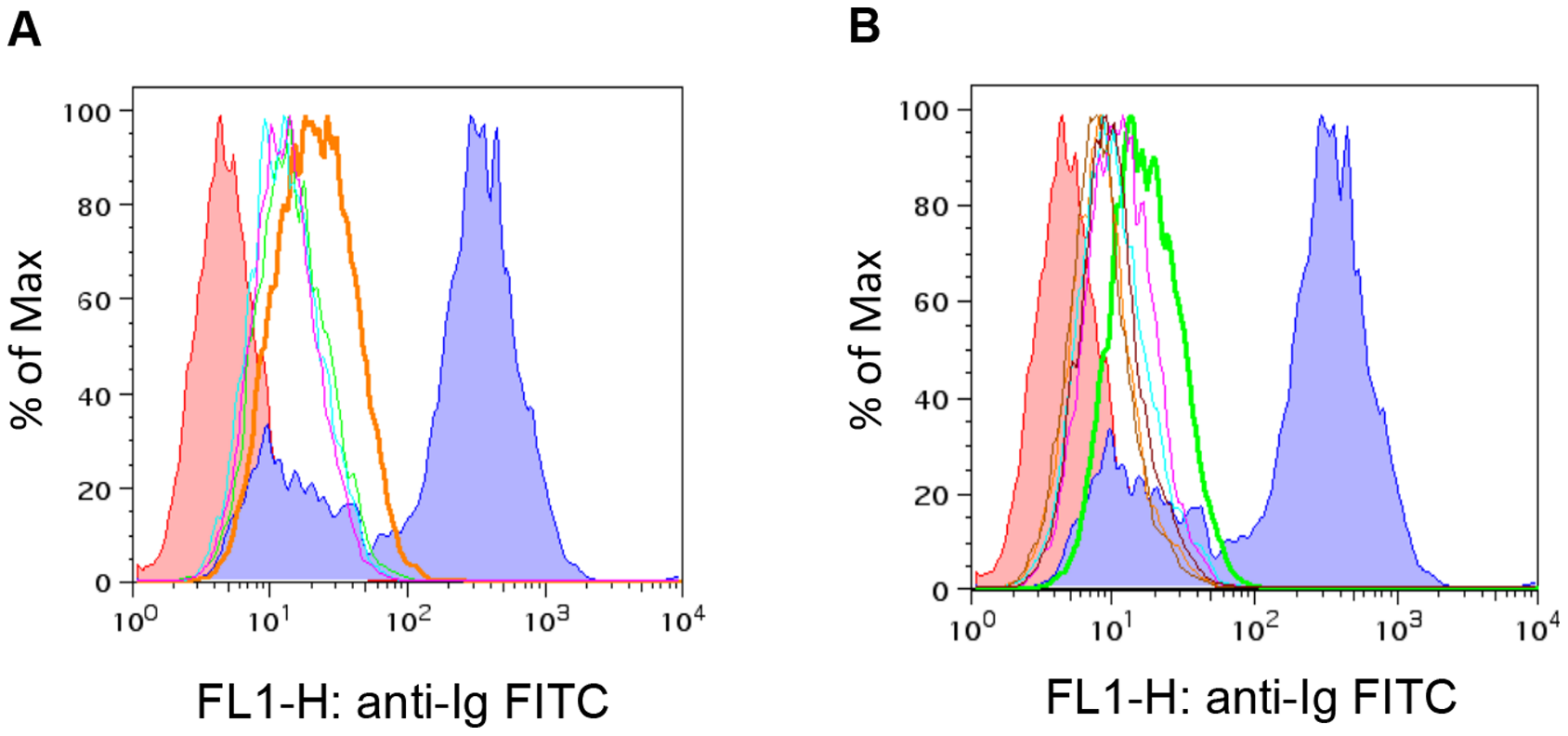
Recognition of PrP on the cell surface. Transgenic DT40 cells expressing PrP on their surface were stained with sera from 4 mice immunized with agPrP+FA (red, green, purple and cyan histograms) (A) or from 6 mice immunized with sPrP+DnaK+FA (red, green, purple, cyan, orange and brown histograms) (B), to evaluate recognition of PrP on the cell surface. Indigo; 6H4 staining, red negative control (secondary antibody only). Differences in the modes of the histograms between agPrP+FA and sPrP+DnaK+FA mice are statistically significant (T-test, P = 0.330).

Surface expression of the prion protein on the DT40 cells was verified by 6H4 binding, which was used as a control. The experimental immune sera tested reacted weakly with surface PrP, however sera from mice immunized with the aggregated form of PrP bound stronger (T-test, P = 0.330) than sera from mice immunized with PrP+DnaK+AgPrP (Figure 5).

#### iii) Cell proliferation assays

Preliminary expression analysis of genes associated with the immune response (namely interleukin 4, intereukin 6 and interferon gamma), in splenic cells harvested from immunized, terminally ill mice, which were cultured in the presence of sPrP suggested the initiation of an humoral immune response in splenocytes originating from mice immunized with sPrP+DnaK+FA, but not from mice immunized with agprP+FA (data not shown). To better characterize the immune response elicited by the immunization, splenocyte proliferation assays were performed.

**Figure 6:**
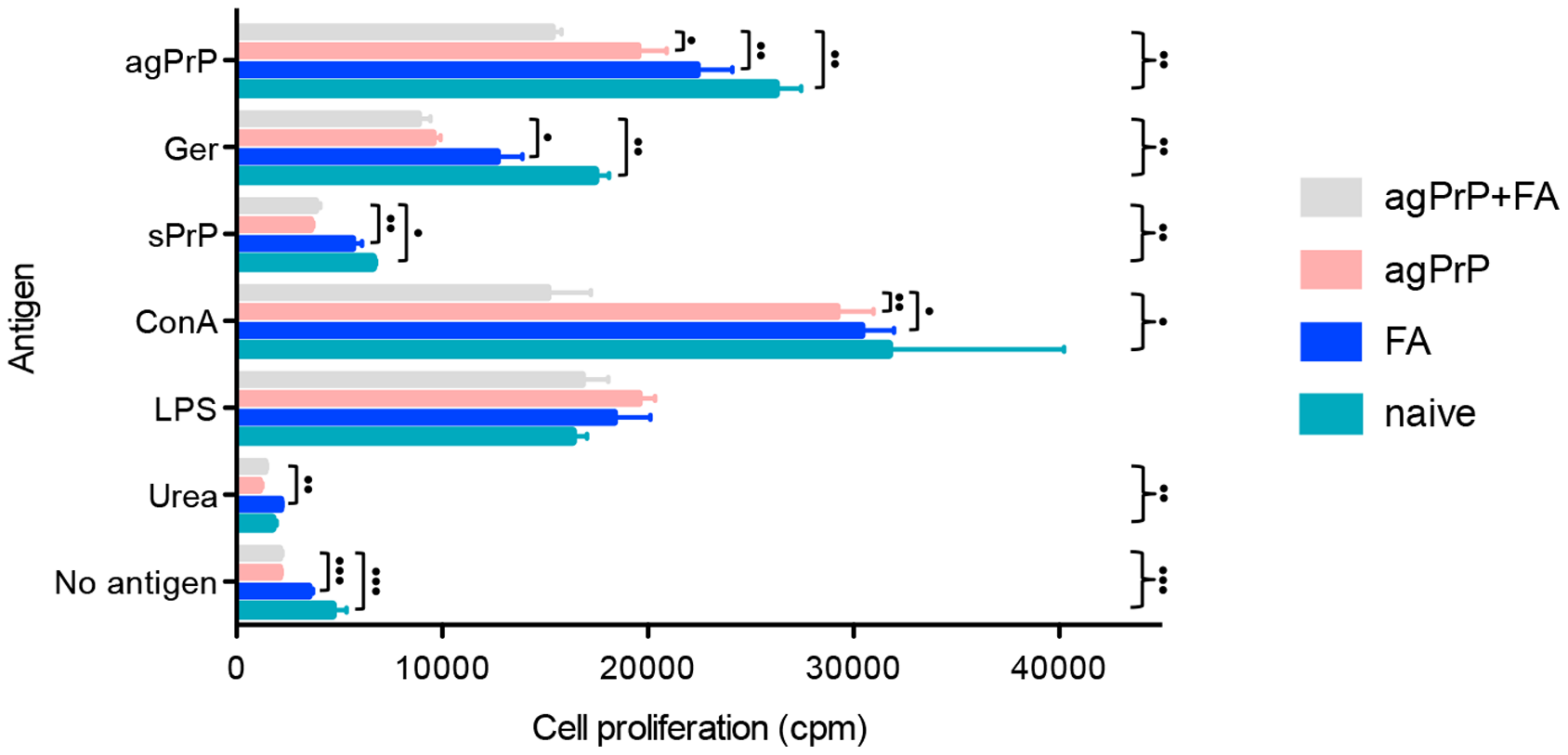
Cell proliferation analysis. Cell proliferation of splenocytes harvested from mice immunized with agPrP+FA, agPrP or FA alone and naive mice cultured in the presence of agPrP, Ger, sPrP (125 µg/ml), ConA (0.5 µg/ml), LPS (5 µg/ml), Urea (0.13 M) or left unstimulated. Each bar corresponds to the average cell count ±SEM. Kruskall-Walliss (}) and Mann-Whitney (]) tests were performed to assess differences between groups. •P<0.05, ••P<0.01, •••P<0.001.

Thus, splenocytes were harvested from naive mice or mice immunized with agPrP and FA alone or agPrP+FA ten days after completion of the immunization protocol. This time point coincides with administration of the infectious agent in the bioassay and being closer to the completion of the immunization protocol, should lead tostronger immune reactions. The splenocytes were cultured in the presence of agPrP, germacrene β-synthase, sPrP, ConA, LPS or urea (Figure 6). Germacrene β-synthase was included in the study as a conformational control since it was prepared in the same bacterial cells and its inclusion bodies were prepared and purified in exactly the same way as agPrP. As a result germacrene β-synthase aggregates should be similar in conformation to agPrP, but differ in their primary structure. Conversely sPrP bears the same primary structure as agPrP, but a different conformation, closer to the native one. ConA and LPS were included as positive controls. Splenocyte proliferation in the presence of ConA is associated with Β-lymphocyte propagation, whereas LPS is associated with T-lymphocyte proliferation. Since sPrP solutions contained a small amount of urea, some splenocytes were grown in the presence of urea, to evaluate any effect low urea concentrations (0.13 M, identical to the ones in the sPrP) cultures may have on cell proliferation.

Splenocytes from mice immunized with agPrP+FA proliferated less than all other splenocytes in the presence of agPrP (Kruskal-Walliss and Mann-Whitney tests), even though the latter was used for the immunization of the mice. Comparing the proliferation data obtained in the presence of agPrP in the cell culture, it appears that, since splenocytes from mice immunized with agPrP or FA alone or even naive mice proliferate more than splenocytes immunized with agPrP+FA, co-administration of agPrP and FA exerts an inhibitory effect on cell proliferation. This effect diminishes as one of the two components is omitted. Analogous results were obtained when the splenocytes were kept in culture in the presence of the similarly prepared germacrene β-synthase, however proliferation was slower in all cultures, compared to the proliferation achieved in the presence of agPrP. In this case, the addition of FA to the immunogen did not further reduce proliferation. AgPrP appears to be a potent stimulator of splenocyte proliferation leading to strong responses; cell proliferation of splenocytes from naive mice proliferate similarly in the presence of agPrP and ConA, whereas a stronger proliferation is obtained with agPrP rather than LPS (Mann-Whitney test, P = 0.05). On the other hand, sPrP induced much slower stimulation rates. This result is consistent with preliminary expression data, in which sPrP failed to initiate an immune response when splenocytes from mice immunized with agPrP were cultured in the presence of sPrP.

#### iv) IHF

Given the interesting, but inconclusive in terms of protection against prion diseases, results of the cell proliferation assays and to better understand the effects immunization with agPrP+FA may have, we sought to determine variations in the splenic microarchitecture and grossly estimate the populations of cells formerly implicated to play a role in propagation of prion infection in naive mice, mice immunized with FA or agPrP alone or with agPrP+FA. These experiments were performed 10 days after completion of the immunization protocol, contemporary to spleen harvest for the cell proliferation assays and aimed at detecting B and T lymphocytes, dendritic cells, macrophages and mature follicular dendritic cells in splenic cryosections. No major aberrations in the positioning and number of B and T lymphocytes, dendritic cells and macrophages were detected (data not shown).

**Figure 7:**
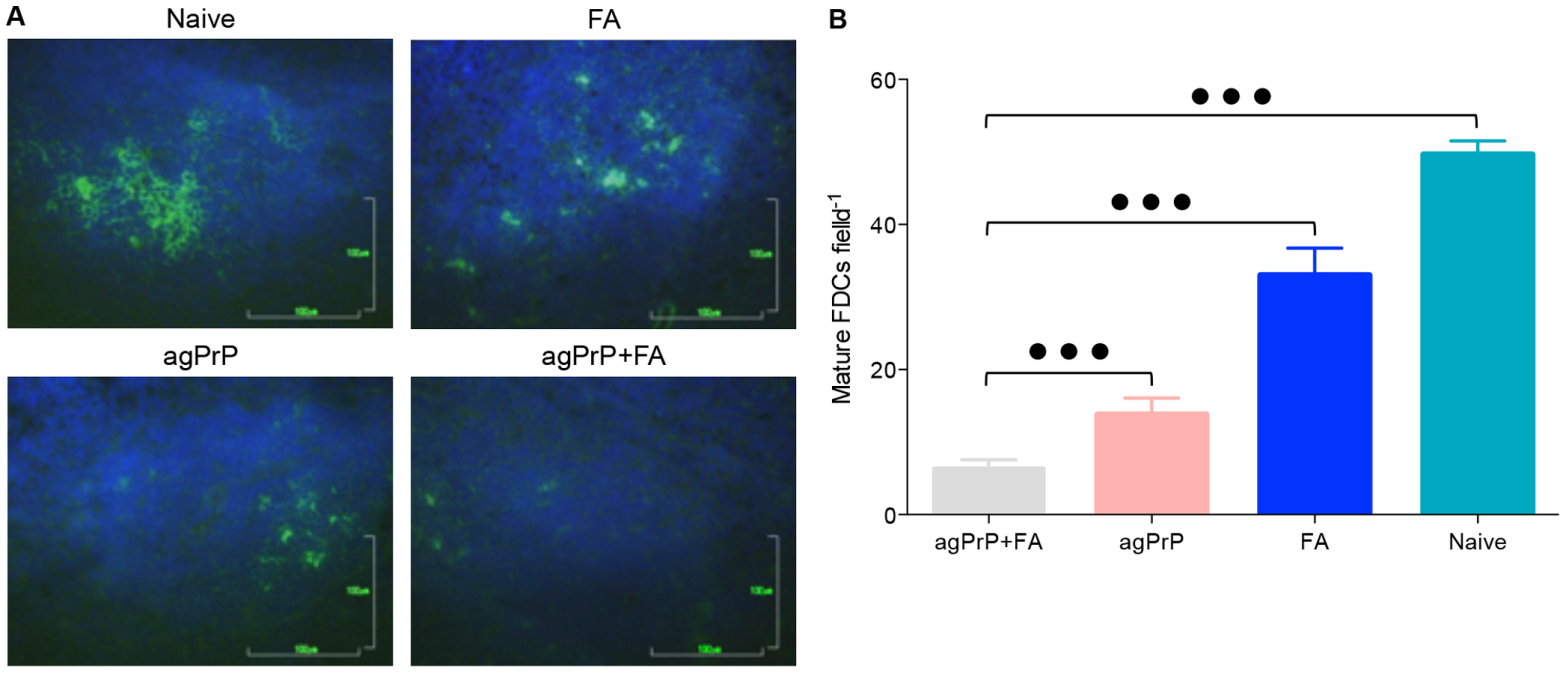
Immunization reduces the number of mature FDCs in the spleens. A. Typical fluorescent microphotographs of mature FDCs in splenic cryosections from naive mice or mice immunized with FA, agPrP and agPrP+FA, following immunostaining with the monoclonal antibody FDC-M1 (green). Bars 100 µm. B. The number of mature FDCs was estimated by counting the mature FDCs in microscopic fields. At least 10 fields from different cryosections of 4 animals from each group were used. The fields were randomly selected using the presence of a germinal center as the only criterion. Each bar corresponds to the mean ±SEM. The differences between the groups are statistically significant (Mann-Whitney test, P<0.0001).

However when mature follicular dendritic cells were visualized with an antibody directed against FDC-M1, a drastic reduction of the cell numbers in mice immunized with agPrP+FA was found. Mature FDCs in ten randomly selected fields containing germinal centers from at least 4 mice from each group were counted by an investigator blinded to the immunization status of each sample. Mice immunized with FA alone displayed a moderate reduction in the population of mature FDCs, whereas mice immunized with agPrP alone and especially agPrP+FA suffered a marked reduction (Figure 7). The differences in cell numbers among the groups are statistically significant (ANOVA, P<0.0001).

## Discussion

Passive immunization with monoclonal antibodies against PrP [21], [35] or the creation of transgenic mice expressing antibodies against PrP [22], provide effective protection against transmissible spongiform encephalopathies, offering proof of principle for prophylaxis. However, the low antigenicity of the prion protein, probably associated with tolerance effects [34], significantly impedes the development of active immunization protocols that would enable a more effective treatment. In our immunization studies we evaluated the antigenicity and possible immunoprotective role against TSEs of several recombinant prion proteins administered on their own or in combination with known immunogenic molecules, trying to circumvent the occurrence of tolerance. Within this frame, immunization of wild-type mice was attempted with a fusion protein consisting of DnaK and mouse prion protein [29], a mixture of recombinant bacterial DnaK and recombinant murine prion protein, a highly purified aggregated recombinant mouse prion protein, and with a partially refolded soluble form of recombinant murine prion protein.

Conjugation of proteins with heat shock proteins is known to increase their antigenicity [36], [37] and chemical cross–linking of the prion protein with DnaK was shown to induce synthesis of antibodies against recombinant PrP, even in wild-type mice [38]. We thus investigated whether immunization with a fusion protein comprising DnaK and PrP would elicit a useful immune response.

Furthermore we investigated the immunization potential of recombinant murine protein administered in non-native conformations. Previous studies in our laboratory demonstrated that administration of the antigen in aggregated rather than soluble form yielded stronger immune responses [39]. Moreover it is known that the ordered, high-concentration delivery of the antigen enhances its immunogenicity [40]. We thus opted to deliver the antigen as inclusion bodies. The exact structure of PrP in the inclusion bodies is not known. However, it has been suggested that proteins embedded in particles are not completely devoid of structural order, maintaining 50–70% of their normal conformation and that inclusion bodies favor the creation of β-pleated sheet [41]. Considering that PrP^Sc^ is insoluble and enriched in β-pleated sheets, it could be argued that within the inclusion bodies, recombinant PrP acquires a conformation which resembles the pathogenic isoform, retaining its insolubility but lacking proteinase K resistance. The soluble form of PrP was produced following gradual dilution of urea in the solution and is the closest conformation to normal included in the study. Still, due to the presence of a chaotropic agent in the solution, its conformation most probably deviates from normal. To enhance the immunogenicity of this form, sPrP was also administered in combination with DnaK.

Challenge of the immunized mice with a mouse adapted scrapie strain, indicated that only immunization with agPrP+FA provided partial protection in the murine model of prion diseases used. Nevertheless, sera from these mice failed to recognize recombinant PrP in ELISA and western blots, as well as PrP^Sc^ and total PrP in brain homogenates from terminally ill mice. On the other hand, sera from mice with sPrP+DnaK+FA produced a strong reaction in both ELISA and western blotting although no protection against the disease was achieved. The ability of antibodies to provide protection against TSEs, has been linked to their capacity to recognize PrP on the cell surface [34]. Thus the protection -or lack of it- despite the absence -or presence- of antibodies recognizing PrP could be explained on the basis of presence of such antibodies in the sera. As expected, sera from mice immunized with sPrP+DnaK+FA failed to react with surface-expressed PrP, but mice immunized with agPrP+FA reacted only mildly. This mild reaction might be associated with an underrepresentation of the PrP reacting clones and could be associated with the observed prolongation of survival interval. On the other hand, this mild reaction could indicate that different mechanisms might be associated with protection.

We first evaluated the possibility that immunization triggered a cellular immune response, which resulted in degradation of PrP^Sc^. However apreliminary study of cytokine expression profile argues against the elicitation of such mechanisms following immunization with agPrP+FA.

The proliferation potential of splenocytes isolated from immunized or naive mice cultured in the presence of different antigens was also used to evaluate the immune response type. Splenocytes from mice immunized with agPrP+FA showed a lower proliferation potential compared to splenocytes from mice that received only the adjuvant, or even naive mice. These data indicate that immunization with agPrP may actually have an immunosuppressant role and that agPrP *per se* may be a potent stimulator of cell proliferation. Since cell proliferation in the presence of LPS is similar in all cultures, whereas cell proliferation of splenocytes from mice immunized with agPrP+FA in the presence of ConA is compromised, it might be argued that the immunosuppression is T-rather than B-lymphocyte associated. Comparison of the cell proliferation data of splenocytes from naive mice in the presence of agPrP, Ger, or sPrP, indicates that stimulation of the proliferation potential is associated with the primary structure of the protein (agPrP induces a stronger reaction than Ger) as well as the conformation (the aggregated form of murine PrP induces a stronger reaction than the soluble form).

To better understand the immunosupressant and protective effect immunization with agPrP+FA has, we attempted an immunohistological analysis of the spleen, giving particular weight to cell types that have been implied to partake an important role in peripheral prion pathogenesis. Histological assessment of the germinal centers did not reveal significant differences between immunized and non-immunized mice. Similarly, differences in the number of B and T lymphocytes and the microarchitecture of the germinal centers or the number of dendritic cells and macrophages were not found. However, the number of mature follicular dendritic cells in the spleens of mice immunized with agPrP+FA was significantly reduced compared to naive mice or mice that had received only the adjuvant. Although administration of agPrP alone led to a reduction of the number of mature FDCs, it was coadministration of agPrP and FA that resulted in the most marked reduction. Similarly, proliferation of splenocytes in the presence of agPrP was slower in splenocytes from mice immunized with agPrP+FA, compared to splenocytes from mice immunized with agPrP alone, indicating that immunization with agPrP and FA has an additive effect on the suppression.

The reduction of the number of mature FDCs in immunized mice could explain both the reduced cell proliferation and the prolongation of survival interval. Mature FDCs act as antigen presenting cells and are important for immunological memory [42], [43]. Although other cell types are involved in antigen presentation, the acute reduction of this cell population could lead to deterioration of antigen presentation thus hampering splenocyte-proliferation. In support of this hypothesis, there is a correlation between cell proliferation and number of mature FDCs in mice groups immunized with the different antigens (Figures 6 and 7).

The reduced number of mature FDCs offers a plausible explanation for the prolongation of survival interval in the mouse model of TSEs. Since mature FDCs play a major role in peripheral prion infection [9], [16], their reduction impedes early PrP^Sc^ propagation, leading to the observed reduction of early splenic PrP^Sc^ accumulation and prolongation of survival interval. The prolongation, rather than full protection against prion diseases, which is also consistent with the accumulation of similar levels of splenic PrP^Sc^ in both control and immunized mice in intermediate (80 days) time points, could be linked either to redundancy phenomena, or to a transient depletion of the mature FDCs population.

Evidence indicates that disease propagation can be achieved even in the absence of mature FDCs and different cell types have been proposed to partake in PrP^Sc^ propagation [10], [11]. Immunization with agPrP should not affect the function of these different cells, thus allowing disease progression with a slower rate. Our survival data could also be the effect of the kinetics of mature FDCs depletion. A study of the kinetics of the depletion of FDCs following immunization with agPrP was not possible, but it may well be that the mature FDCs population slowly returns to its pre-immunization, PrP^Sc^-propagation-permissive status. Of note, prolongation of the mature FDCs depletion by repetitive administration of anti-lymphotoxin-β receptor Ig has a minor impact on disease progression indicating that in the absence of mature FDCs PrP^Sc^ can be peripherally amplified in other cells as well [44].

The mechanism governing depletion of mature FDCs is not clear, however it could be argued that similarly to anti-lymphotoxin-β receptor Ig administration:i) FDCs return to a non-differentiated premature condition, which affects phenotype and function, ii) FDCs undergo apoptotic death in the absence of the necessary cytokine signaling, iii) the cytokine gradient essential for the organization of germinal centers is modified and as a consequence FDCs disperse [45]. Typical germinal centers were identified in the splenic preparations from immunized mice, indicating that most probably their organization is not affected by immunization with agPrP. Moreover, B-lymphocytes, which secrete lymphotoxin-β and are most important for the maintenance of mature FDCs, were detected, although their lymphotoxin-β-secretion function could not be evaluated. Furthermore, the characteristic morphological alteration accompanying apoptotic death was not observed, suggesting that at least 10 days after the immunization protocol was completed, FDCs did not undergo apoptotic death. Thus, our data point towards an unidentified FDCs dedifferentiation mechanism.

Immunization with agPrP+FA provided partial protection in a mouse model of prion diseases, associated with depletion of mature FDCs and with the development of antibodies recognizing prion protein on the cell surface. Depletion of mature FDCs following agPrP immunization was mediated via an as yet obscure mechanism probably associated with the cells’ dedifferentiation. To our knowledge this is the first report that immunization with a recombinant form of PrP leads to depletion of mature FDCs and although further studies are required, this observation could form the basis for a novel therapeutic approach for spongiform encephalopathies and other FDC-associated ailments [46].

## Supporting Information

Figure S1 **Preparation of sPrP.** A. Western blot analysis of the purified recombinant murine PrP with the monoclonal antibody 6H4 (A) and SDS-PAGE of the purified recombinant murine PrP (B).
(TIF)

Figure S2 **Accumulation of PrP^Sc^ in the brain of terminally ill mice.** 2.5 mg brain equivalent from one mouse per group were enriched in PrP^Sc^ and blotted with 6H4. Similar amounts of PrP^Sc^ were detected in all groups.
(TIF)

Figure S3 **Neuropathological and immunohistochemical evaluation of terminally ill mice.** A. Haematoxylin/eosin staining and B. GFAP immunohistochemistry from cerebellar sections of terminally ill mice. Mice from all groups display similar degrees of neuropathology and astrocytosis.
(TIF)

Figure S4 **Serum from agPrP+DnaK mice does not recognize PrP in western blots.** A. 2.5 mg brain equivalent from terminally ill mice were blotted with 6H4 (lanes 1, 2) or serum from a mouse immunized with agPrP (lanes 3, 4), prior (lanes 1, 3) or ensuing (lanes 2, 4) PrP^Sc^ enrichment. B. sPrP (1 µg) was blotted with serum from a mouse immunized with sPrP+DnaK.
(TIF)
