# Supplementary figures and images for: Immunization with Recombinant Prion Protein Leads to Partial Protection in a Murine Model of TSEs through a Novel Mechanism
Source: PLoS One. 2013 Mar 15;8(3):e59143. doi: 10.1371/journal.pone.0059143 (PMC3598700; doi:10.1371/journal.pone.0059143)

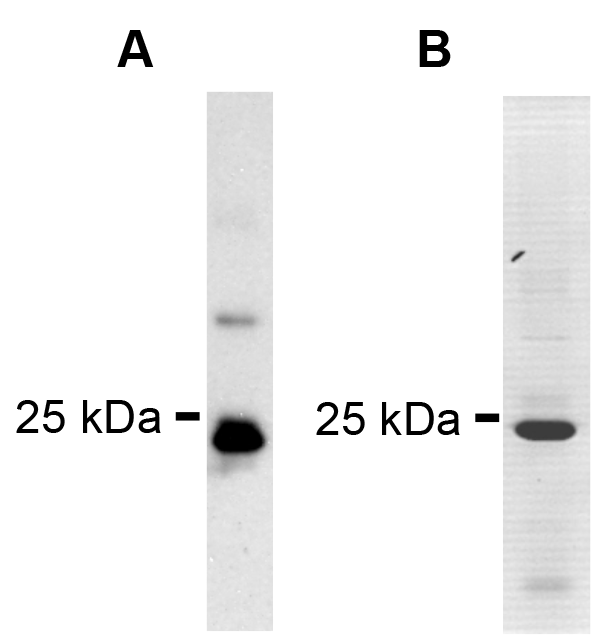

Supplement: Figure S1 — Preparation of sPrP. A. Western blot analysis of the purified recombinant murine PrP with the monoclonal antibody 6H4 (A) and SDS-PAGE of the purified recombinant murine PrP (B). (TIF) [file pone.0059143.s001.tif]

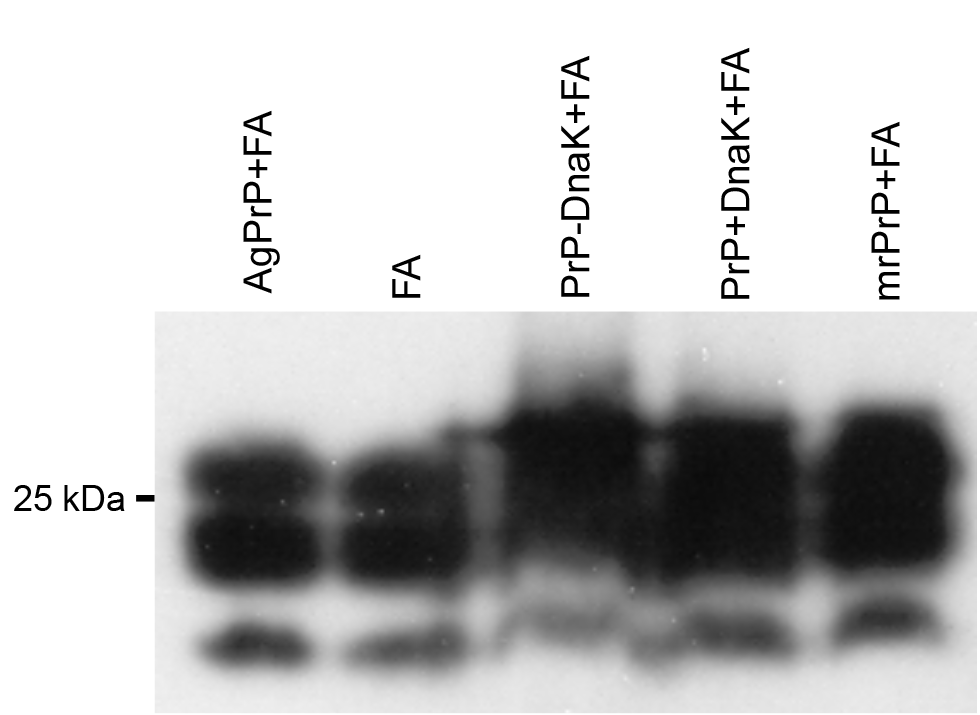

Supplement: Figure S2 — Accumulation of PrPSc in the brain of terminally ill mice. 2.5 mg brain equivalent from one mouse per group were enriched in PrPSc and blotted with 6H4. Similar amounts of PrPSc were detected in all groups. (TIF) [file pone.0059143.s002.tif]

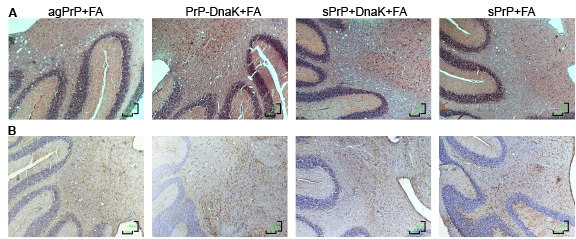

Supplement: Figure S3 — Neuropathological and immunohistochemical evaluation of terminally ill mice. A. Haematoxylin/eosin staining and B. GFAP immunohistochemistry from cerebellar sections of terminally ill mice. Mice from all groups display similar degrees of neuropathology and astrocytosis. (TIF) [file pone.0059143.s003.tif]

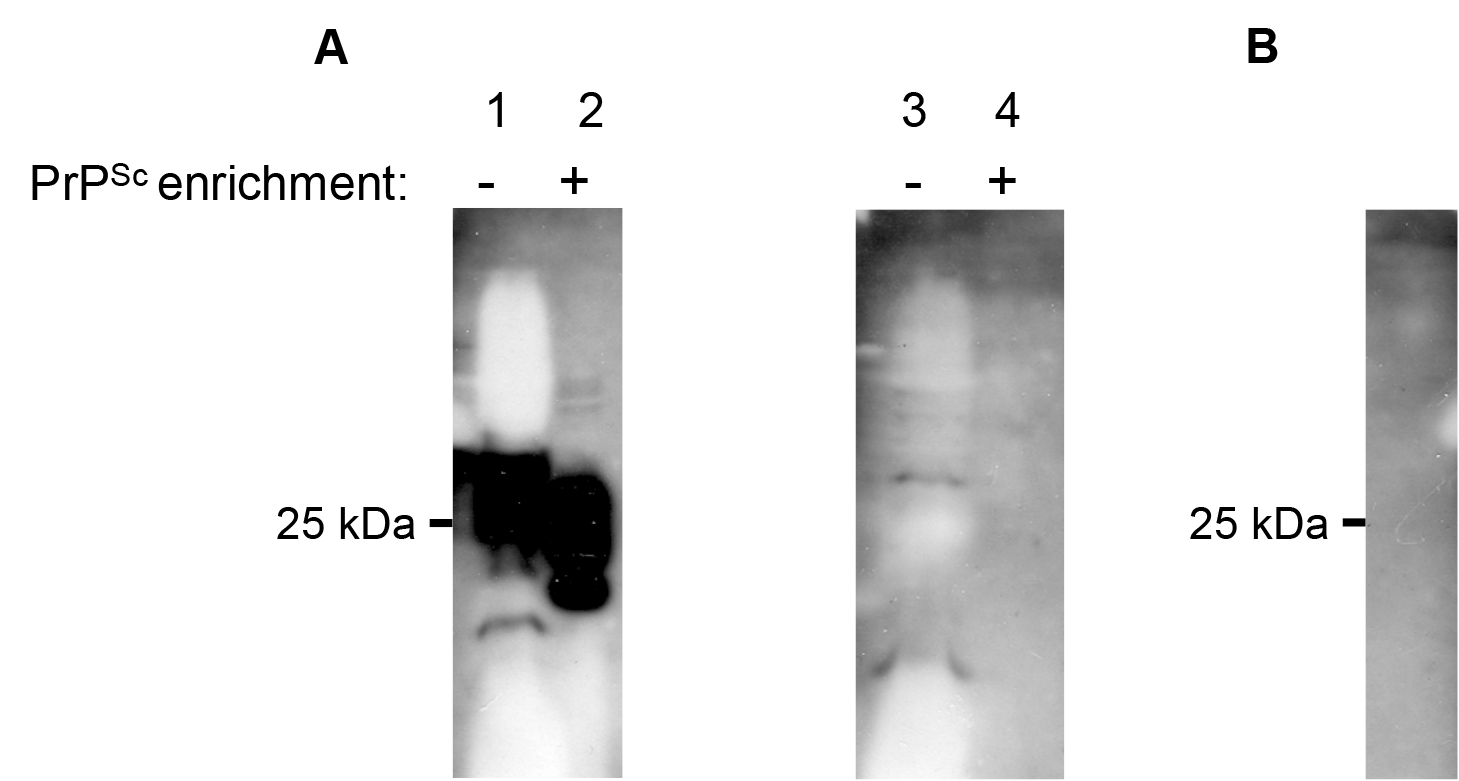

Supplement: Figure S4 — Serum from agPrP+DnaK mice does not recognize PrP in western blots. A. 2.5 mg brain equivalent from terminally ill mice were blotted with 6H4 (lanes 1, 2) or serum from a mouse immunized with agPrP (lanes 3, 4), prior (lanes 1, 3) or ensuing (lanes 2, 4) PrPSc enrichment. B. sPrP (1 µg) was blotted with serum from a mouse immunized with sPrP+DnaK. (TIF) [file pone.0059143.s004.tif]
